# Platelet-derived growth factor receptor-β, carrying the activating mutation D849N, accelerates the establishment of B16 melanoma

**DOI:** 10.1186/1471-2407-7-224

**Published:** 2007-12-12

**Authors:** Shioto Suzuki, Carl-Henrik Heldin, Rainer Lothar Heuchel

**Affiliations:** 1 Ludwig Institute for Cancer Research, Uppsala Branch, Uppsala, Sweden; 2 Department of Molecular and Cellular Pathology, Kanazawa University Graduate School of Medical Science, Kanazawa, Ishikawa, Japan

## Abstract

**Background:**

Platelet-derived growth factor (PDGF)-BB and PDGF receptor (PDGFR)-β are mainly expressed in the developing vasculature, where PDGF-BB is produced by endothelial cells and PDGFR-β is expressed by mural cells, including pericytes. PDGF-BB is produced by most types of solid tumors, and PDGF receptor signaling participates in various processes, including autocrine stimulation of tumor cell growth, recruitment of tumor stroma fibroblasts, and stimulation of tumor angiogenesis. Furthermore, PDGF-BB-producing tumors are characterized by increased pericyte abundance and accelerated tumor growth. Thus, there is a growing interest in the development of tumor treatment strategies by blocking PDGF/PDGFR function. We have recently generated a mouse model carrying an activated PDGFR-β by replacing the highly conserved aspartic acid residue (D) 849 in the activating loop with asparagine (N). This allowed us to investigate, in an orthotopic tumor model, the role of increased stromal PDGFR-β signaling in tumor-stroma interactions.

**Methods:**

B16 melanoma cells lacking PDGFR-β expression and either mock-transfected or engineered to express PDGF-BB, were injected alone or in combination with matrigel into mice carrying the activated PDGFR-β (D849N) and into wild type mice. The tumor growth rate was followed and the vessel status of tumors, i.e. total vessel area/tumor, average vessel surface and pericyte density of vessels, was analyzed after resection.

**Results:**

Tumors grown in mice carrying an activated PDGFR-β were established earlier than those in wild-type mice. In this early phase, the total vessel area and the average vessel surface were higher in tumors grown in mice carrying the activated PDGFR-β (D849N) compared to wild-type mice, whereas we did not find a significant difference in the number of tumor vessels and the pericyte abundance around tumor vessels between wild type and mutant mice. At later phases of tumor progression, no significant difference in tumor growth rate was observed between wild type mice and mutant mice, although the pericyte coverage was higher around tumor vessels from mutant mice.

**Conclusion:**

Our findings suggest that the activated PDGFR-β (D849N) in the host animal increased the total vessel area and the average vessel surface even in PDGF-negative tumors, resulting in a shorter lag phase during tumor establishment.

## Background

Platelet-derived growth factor (PDGF)-BB is a homodimeric growth factor with a broad range of target cells, notably mesoderm-derived cells, such as pericytes and mesangial cells, but also ectoderm-derived glial cells and neurons. PDGF-BB binds to two distinct receptor tyrosine kinases denoted PDGF receptor (PDGFR)- α and – β [1].

PDGF-BB and PDGFR-β are mainly expressed in the developing vasculature, where PDGF-BB is produced by endothelial cells which attract PDGFR-β expressing mural cells, i.e. pericytes and vascular smooth muscle cells [2-4]. Knockout studies of PDGF-B or PDGFR-β identified pericyte deficiency of the microvasculature as a main phenotype, resulting in lethal hemorrhage and edema at late gestation [5-7].

PDGF-BB is produced by most types of solid tumors, inducing stroma formation and neovascular response in order to secure sufficient supply of nutrients and oxygen [8]. Furthermore, it has been shown that PDGFR-β expression by pericytes is necessary for their recruitment to tumor vessels and that extracellular retention of PDGF-BB produced by the tumor endothelium is required for the recruitment of adequate numbers of pericytes, as well as for proper integration of pericytes into the vascular wall [9]. A recent study demonstrated that pericyte density correlates inversely with the effect of antiangiogenic therapy [10]. Using mouse models it was shown that inhibition of PDGF signaling reduces interstitial fluid pressure in tumors, and thereby enhances the effect of chemotherapy [11,12]. Together, these examples suggest potential benefits of targeting PDGFR-β in the treatment of tumors.

Recently, PDGF receptor signaling was shown to be perturbed by mutations within the activation loop of PDGFR-α in a subset of wild-type c-kit expressing gastrointestinal stromal tumors (GISTs) [13]. One of these mutations, D842V, resulted in receptors displaying ligand-independent receptor phosphorylation and activation of downstream signaling molecules [14]. We have recently generated a mouse model carrying a mutation in the corresponding amino acid residue in the activation loop of the murine PDGFR-β; namely an exchange of asparagine for aspartic acid at amino acid position 849 (D849N). This mutation did not cause any tumor formation in homozygous mutant mice, despite the fact that this mutation also caused ligand-independent receptor autophosphorylation [15]. This elevated basal phosphorylation resulted in a dramatically increased ligand-independent anti-apoptotic signaling and migration, as well as a ligand-dependent increase in proliferation of D849N-mutant mouse embryonic fibroblasts. This prompted us to investigate how a host environment carrying a D849N-activated PDGFR-β would influence tumor formation *in vivo*.

In the present study, we show that a stromal environment with the D849N-mutant PDGFRβ favored earlier establishment of PDGF-BB expression-negative tumors up to a size of 0.3 cm^3^, due to increased vascularization, characterized by increased total vessel area and average vessel surface. Above a tumor size of 0.3 cm^3^, the tumor growth rate was similar in wild type and D849N-mutant mice.

## Methods

### Cell culture

B16F10 melanoma cells stably transfected with either empty expression vector (mock) or vector containing an expression cassette for PDGF-B were cultured in Dulbecco's modified Eagle's medium (DMEM) supplemented with 10% fetal bovine serum and antibiotics [16].

### Animals

Generation of D849N-mutant mice carrying an activating mutation in the PDGFR-β have been described earlier [15]. Mutant and control mice were in identical C57Bl/6 strain background. All animal experiments were approved by the local ethical committee and performed according to the United Kingdom Coordinating Committee on Cancer Research guidelines [17].

### Tumor formation assay

B16 melanoma cells (1 × 10^6 ^mock-transfected or PDGF-BB expressing cells) were injected subcutaneously in 100 μl PBS on both sides of the dorsal region of mice anesthetized with isoflurane. The tumor size was obtained by daily measurements of the length and width of the tumor nodules with calipers. The tumor volume was calculated as 3.14/6 × length × width^2^. Tumors were removed surgically from CO_2_-sacrificed mice at maximum tumor size of 0.8–1.0 cm^3 ^and fixed in paraformaldehyde overnight. After embedding tumors in paraffin, sections were cut at 4-μm thickness onto Superfrost Plus slides (Histolab) for Hematoxylin and Eosin (H & E) staining or immunostaining.

### Matrigel injection

Matrigel (BD Biosciences, #354248, high concentration; 150 μl) was mixed by pipetting with 1 × 10^6 ^B16 cells resuspended in 150 μl of PBS, or 150 μl of PBS alone. Matrigel mixes (300 μl) were injected subcutaneously on both sides of the ventral region of wild type and mutant (D849N) mice under isoflurane anesthesia. Matrigel plugs were harvested at the indicated time points and fixed in paraformaldehyde overnight. After embedding matrigel plugs in paraffin, sections were cut at 4-μm thickness onto Superfrost Plus slides.

### Histochemistry and immunohistochemistry

For immunohistochemistry, deparaffinized sections were pretreated by boiling in 10 mM citrate buffer (pH 6.0) for 2 × 7 min at 750 W in a microwave oven. Tissue peroxidase activity was quenched by incubation in 3% H_2_O_2 _in PBS for 10 min, followed by blocking in 20% serum of the secondary antibody species. For double staining of pericytes and endothelial cells, anti- α smooth muscle actin (ASMA) monoclonal antibodies (1 μg/ml; clone 1A4, Dako) and goat-anti-mouse CD31/platelet/endothelial cell adhesion molecule 1 (PECAM-1) antibodies (1 μg/ml sc-1506; Santa Cruz Biotechnology) were used consecutively. Macrophages were identified with rat anti-mouse F4/80 antibodies (1:100, MCAP497, clone A3-1, Serotec). Omission of primary antibody was used as negative control. Primary antibodies for ASMA, CD31 and F4/80 were detected using either biotinylated rabbit-anti-mouse antibodies (1:500, E0354, DAKO) visualized by Vectastain ABC-HRP kit (1:100, Vector Laboratories), or biotinylated rabbit-anti-goat antibodies (1:500, E0466, DAKO) visualized by Vectastain ABC-AP kit (1:100, Vector Laboratories), or biotinylated goat-anti-rat antibodies (10 μg/ml, BA9400, VECTOR) visualized by Vectastain ABC-HRP kit (1:100, Vector Laboratories) respectively, according to the manufacturer's instructions. Mayer's hematoxylin was used as counterstain. Using a Leica microscope (DM4000) equipped with a Leica DFC camera and Qwin software, several parameters, such as the total vessel surface, viable tumor area, number of vessels and pericytes and perimeter of vessels, were calculated. All immunohistochemical analyses were performed on 20 high-power fields per tumor. Only viable tumor area was examined. The ratio of total vessel area per tumor was defined as the total surface of vessel area divided by the area of viable tumor. The average vessel area was defined as total vessel area divided by the total number of vessels. The number of vessels per high-power field was defined as total number of vessels divided by the number of analyzed fields (n = 20). The numerical density of pericytes per perimeter of vessel was defined as the number of pericyte nuclei associated with tumor vessels divided by the sum of vessel perimeter.

### Statistical analysis

Statistical analysis was performed using ANOVA. *P *< 0.05 was considered as statistically significant.

## Results

### PDGF-BB expression increases tumor vascularization in wild type and D849N-mutant mice but has no further increasing effect on pericyte coverage in D849N-mutant mice

**Figure 1:**
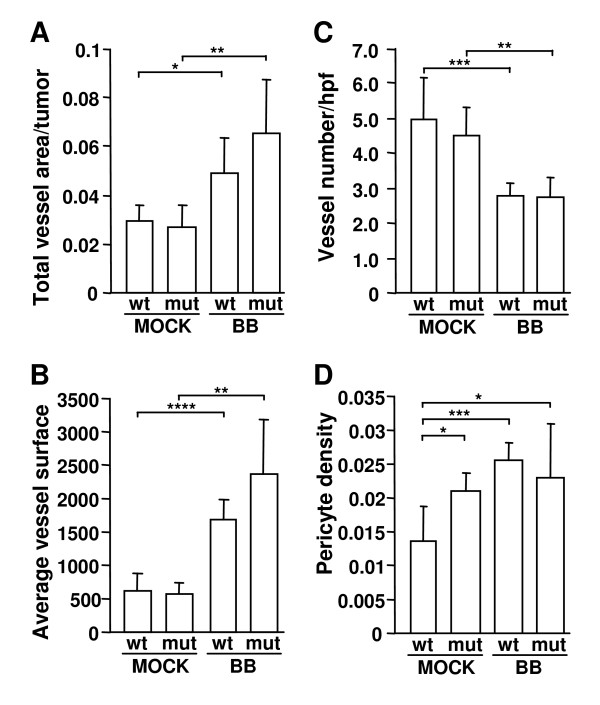
**PDGF-BB expression increases vascularization of B16 tumors**. (A)-(D): Mock-transfected B16 melanoma cells, lacking PDGF-BB expression (Mock) and PDGF-BB expressing B16 melanoma cells (BB) were subcutaneously injected on both sides of the midline of the lower dorsal abdomen of wild type (wt) or D849N-mutant mice (mut). Six tumors were analyzed for each experimental combination. When the B16 melanomas had reached 0.8 cm^3^, they were removed and analyzed for ratio of total vessel area/viable tumor (A), average vessel surface (B), number of vessels per high-power field (C) and numerical density of pericytes per perimeter of vessel (D). Asterisks indicate statistical significance (*, p < 0.05; **, p < 0.01; ***, p < 0.001; ****, p < 0.0001) by Student's *t*-test.

It has been shown that the ectopic expression of PDGF-BB by subcutaneously injected melanoma cells has a positive effect on tumor vascularization [16]. Therefore, we first analyzed the effect of PDGF-BB-expression on the vascular compartment in large (0.8 cm^3^) B16 tumors in wild type mice and D849N-mutant mice (n = 6 tumors for each experimental group). Staining for CD31 identified endothelial cells, whereas perivascular cells/pericytes were identified by staining for ASMA. In both wild type and D849N-mutant mice, the ratio of vessel area/tumor area was higher for PDGF-BB-expressing B16-tumors, compared to mock-transfected B16-tumors (wild type p < 0.05, D849N-mutant p < 0.01) (Fig. 1A). However for PDGF-BB expressing tumors, there was only a tendency (p = 0.1371) towards an increased ratio of vessel area/tumor area in D849N-mutant mice (0.065 +/- 0.022) compared to wild type mice (0.049 +/-0.014) (Fig. 1A). The average vessel surface, defined as the total vessel area divided by the number of vessels, was increased in PDGF-BB-expressing compared to wild type B16 tumors, grown in wild type (p < 0.0001), as well as D849N-mutant mice (p < 0.01) (Fig. 1B). Furthermore, the average vessel surface of PDGF-BB-expressing tumors was slightly higher in D849N-mutant mice (2362 +/- 824) than in wild-type mice (1679 +/- 301), although the difference was not statistically significant (p = 0.0649) (Fig. 1B). On the other hand, the number of vessels per high-power field in PDGF-BB-expressing tumors was decreased in wild type mice (p < 0.001) and D849N-mutant mice (p < 0.01) compared to wild type tumors (Fig. 1C).

**Figure 2:**
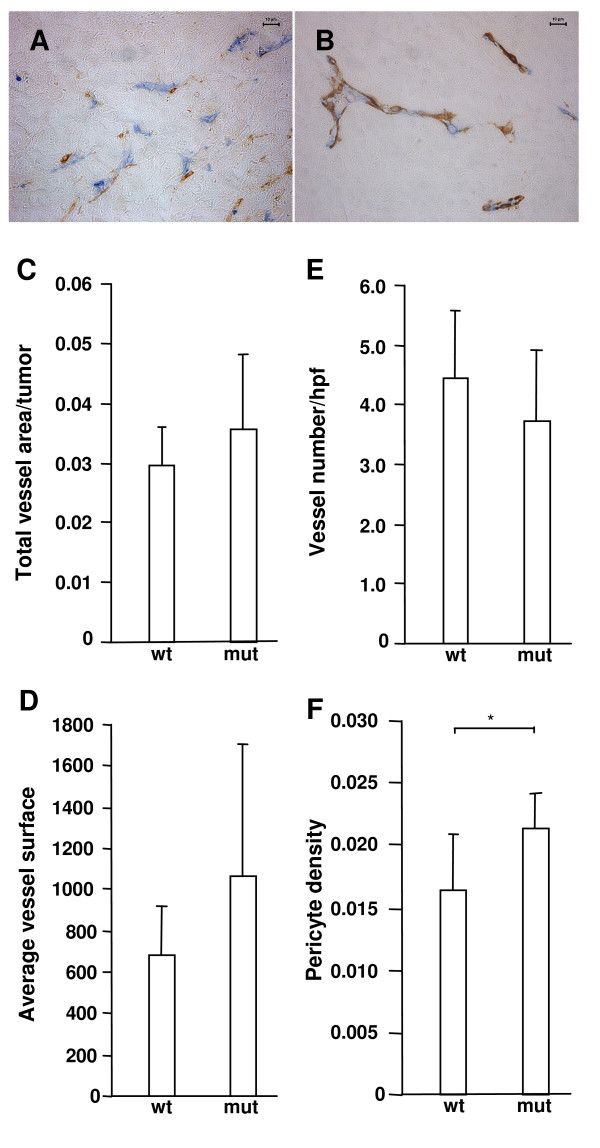
**D849N-mutant PDGFR-beta results in increased pericyte coverage of vessels**. B16 melanoma mock cells, which do not produce PDGF-BB, were subcutaneously injected and processed as described in the legend of figure 1. Double immunohistochemical staining for endothelial cells (CD31, blue) and perivascular cells (α-smooth muscle actin, brown) in B16 melanoma is shown. Please note the small vessels (blue) covered by few pericyte (brown) in tumors grown in wild type mice (A) compared to larger vessels surrounded by multiple perivascular cells (brown) in tumors grown in D849N-mutant mice (B). Tissue sections were analyzed for total vessel area (C), average vessel surface (D), number of vessels per high-power field (E) and numerical density of pericytes per vessel perimeter (F). Asterisk indicates statistical significance (*, p < 0.01) by Student's *t*-test.

Since pericyte recruitment is a well-documented effect of PDGF signaling, we calculated the pericyte density of vessels as the total number of pericytes divided by the total perimeter of vessels. PDGF-BB-expression significantly increased the pericyte density in B16 tumors (0.8–1.0 cm^3^) grown in wild type mice (p < 0.001) (Fig. 1D). Such an effect was not observed for tumors grown in D849N-mutant mice. Instead, in comparison to the wild type B16 tumors grown in wild type mice, those grown in D849N-mutant mice generally displayed increased pericyte numbers per vessel perimeter, independent of tumoral PDGF-BB expression (p < 0.05) (Fig. 1D). We confirmed these initial findings in another experimental series, in which we only used mock-transfected B16 melanoma cells (twelve tumors each from wild type and D849N-mutant mice). Again, increased pericyte density of B16 tumor vasculature was the only statistically significant difference between tumors grown in wild type and in D849N-mutant mice (Fig. 2F).

### Activated PDGFR-β accelerates B16 tumor growth

**Figure 3:**
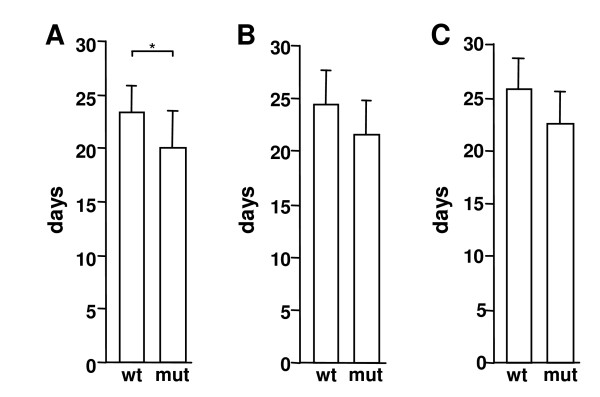
**Accelerated tumor establishment in D849N-mutant mice**. The average time required until injected tumor cells reached volumes of 0.3 (A), 0.5 cm^3 ^(B) and 0.8 cm^3 ^(C), respectively, are shown. Asterisk indicates statistical significance (*, p < 0.05) by Student's *t*-test.

In order to test if the increased pericyte coverage has any functional consequences on tumor development, we measured the tumor growth rate, i.e. the number of days needed from the injection time point until tumors had reached sizes of 0.2, 0.3, 0.5, or 0.8 cm^3 ^in eight wild type and eight D849N-mutant mice. Tumors in D849N-mutant mice grew significantly faster predominantly during the early establishment of the tumor, i.e. until it had reached a volume of 0.3 cm^3^. This took 20.1 days (19.4 for 0.2 cm^3^) in mutant mice, compared to 23.4 days (22.5 for 0.2 cm^3^) in wild type mice (p = 0.047<0.05 for 0.3 cm^3^; p = 0.0216 for 0.2 cm^3^; Fig. 3A and data not shown). We also observed slightly increased growth rates at later stages, although this was not statistically significant (0–0.5 cm^3^, p = 0.097; 0–0.8 cm^3^, p = 0.065; Fig. 3B and Fig. 3C).

### The mutant PDGFR-β causes increased tumor vascularization, supporting early tumor establishment

**Figure 4:**
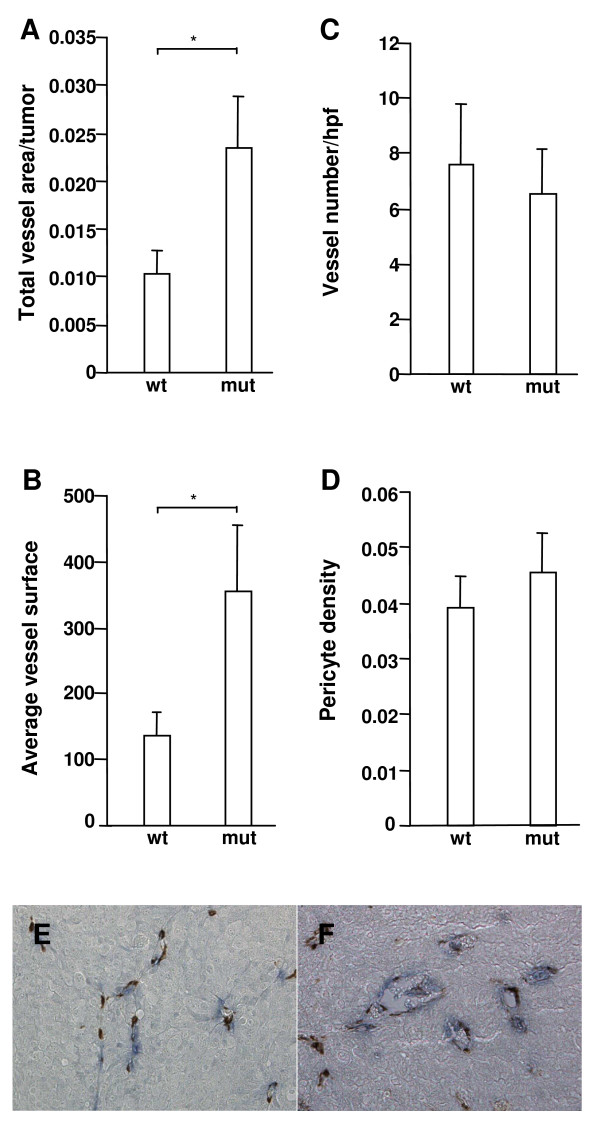
**D849N-mutant PDGFR-beta increases the ratio of vessel area per tumor and average vessel area**. Matrigel mixed with mock-transfected B16 melanoma cells was subcutaneously injected into mice and processed after 8 days as described in the legend of figure 2. Tissue sections were analyzed for the ratio of total vessel area/tumor (A), average vessel surface (B), number of vessels per high-power field (C) and numerical density of pericytes per perimeter of vessel (D). Asterisks indicate statistical significance (*, p < 0.01) by Student's *t*-test. Double immunohistochemical staining for vessels (CD31, blue) and perivascular cells (alpha-smooth muscle actin, brown) identify significantly larger vessels in B16 melanoma/matrigel plugs grown in wild type mice (E) compared to D849N-mutant mice (F) (the same magnification was used in Fig. 4E and Fig. 4F).

Based on the above results one might speculate that the increased pericyte coverage of the tumor vasculature observed in D849N-mutant mice could cause accelerated establishment of tumors due to improved vessel function. However, it is technically very difficult to follow the early stages of tumor establishment from subcutaneously injected cells. We therefore mixed tumor cells with matrigel before injection in order to make it easier to localize the early stages of tumor establishment [18]. Matrigel plugs were removed 8 (a) or 10 (b) days after injection, and the tumor vessel status was analyzed as described before (a: n = 5 plugs each from wild type and D849N-mutant mice; b: n = 9 plugs from wild type mice, n = 13 plugs from D849N-mutant mice). At both time points, the ratio of vessel area per tumor was significantly higher in mutant (a = 2.35%, b = 3.35%) than wild type mice (a = 1.03%, b = 1.59%) (Fig. 4A and data not shown). Furthermore, the average vessel area was (a) 2.62- and (b) 3.24-times larger in mutant mice than in wild type mice (a, p < 0.01; b, p < 0.01; Fig. 4B and data not shown). Surprisingly, no difference in the number of vessels and pericyte density could be observed at this early time point of tumor development (Fig. 4C and 4D, and data not shown). These observations indicated that the early establishment of tumors in mice with D849N-mutant PDGFR was not caused by increased pericyte coverage, but by increased vascularization (ratio of vessel area per tumor and average vessel area) and thus improved blood supply.

### D849N-mutant ASMA-positive cells are characterized by accelerated matrigel invasion

**Figure 5:**
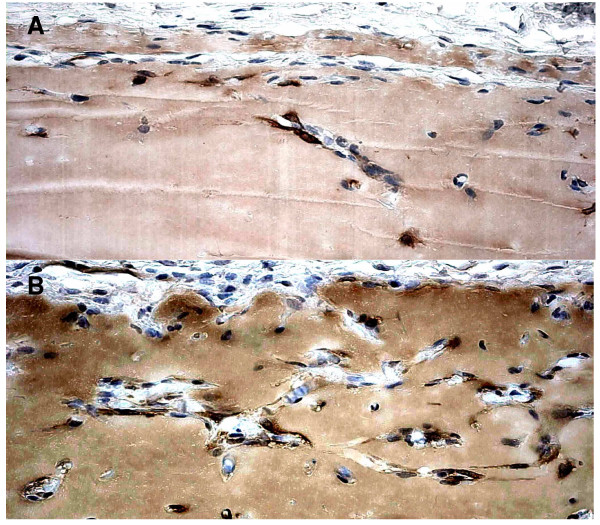
**Immunohistochemical staining for α-smooth muscle actin**. Immunohistochemical staining for α-smooth muscle actin (ASMA, brown) showing clearly more ASMA-positive cells in the matrigel plugs of mice carrying the activated PDGFR-beta (B), compared wild type mice (A) (the same magnification was used in Fig.5A and Fig.5B).

PDGFR-β-expressing cells, such as macrophages, fibroblasts and perivascular cells, have been shown to pave the way for new vessels [1,19]. Since we observed a strongly increased ligand-independent migration in D849N-mutant mouse embryonic fibroblasts [15], we tested if stromal cells of the mutant mouse would invade matrigel plugs more efficiently than those from wild type mouse. matrigel plugs harvested 6 days after injection from wild type (n = 10) or mutant mice (n = 9) were equally positive for the monocyte/macrophage marker F4/80 (data not shown). However, we found more ASMA-positive cells in the matrigel plugs of mutant mice, indicating a more invasive/motile stromal compartment in mice carrying the activated PDGFR-β (Fig. 5).

## Discussion

The crucial importance of PDGF-B and PDGFR-β for vessel formation and maturation has been unambiguously demonstrated by gene targeting experiments [4]. In our study, ectopic PDGF-BB expression by B16 melanoma cells correlated with increased vascularization and pericyte coverage in established, large (0.8–1.0 cm^3^) tumors. This is in accordance with previous studies indicating that PDGF-BB induces more mature vessels with higher pericyte density [9,10,16]. Interestingly, we found the pericyte density elevated in mice carrying an activating mutation (D849N) in PDGFR-β, independent of ectopic PDGF-BB expression, supporting earlier observations that this mutation causes significant receptor signaling in the absence of ligand, or greatly increases sensitivity towards very low amounts of ligand [15]. Since the pericyte coverage of the tumor vasculature in D849N-mutant mice was autonomously elevated, we continued our experiments with B16 melanoma cells lacking PDGF-BB expression. In several mouse tumor models, it has been shown that paracrine stimulation of PDGFR-β is associated with increased tumor growth rate [16,20]. In agreement with this, we found that tumors grew faster in mice with activated PDGFR-β, compared to wild type mice. However, this was statistically significant only during tumor establishment, i.e. until a volume 0.2 to 0.3 cm^3^. In order to investigate the influence of the host stroma on early tumor establishment, we mixed B-16 melanoma cells with matrigel. In contrast to the situation in large tumors (0.8–1.0 cm^3^), there was no difference in pericyte density, but the tumors grown in D849N-mutant mice were characterized by significantly increased vascularization. Therefore, the growth advantage during tumor establishment is most probably due to increased blood supply. Experimental angiogenesis is characterized by a sequential invasion of subcutaneous matrigel plugs by monocyte/macrophages, fibroblasts, pericytes and endothelial (precursor) cells [19]. Although we did not see any difference in macrophage staining in subcutaneous matrigel plugs lacking tumor cells (data not shown), we found that ASMA-positive stromal cells in D849N-mutant mice more efficiently invaded matrigel plugs. This implies that a more active stromal compartment probably drives the improved vascularization during tumor establishment in mice carrying an activated PDGFR-β. On the other hand, it seems unlikely that an enhanced direct interaction between stromal fibroblasts and tumor cells could account for the earlier tumor establishment in D849N-mutant mice, since there was no difference in proliferation of B16 melanoma cells co-cultured with either irradiated wild type or irradiated D849N-mutant mouse embryonic fibroblasts *in vitro *(data not shown). However, we cannot exclude an increased participation of circulating hemangioblasts (hematopoietic/endothelial precursor cells) in early tumor vascularization in D849N-mutant mice. Recently, increased PDGFR-β signaling due to transgenic overexpression of PDGF-B, or the D849N-mutant PDGFR-β was shown to promote endothelial cell differentiation of hemangioblasts *in vivo *at the expense of hematopoietic differentiation [21].

## Conclusion

In summary, we have found two different effects of D849N-mutant PDGFR-β signaling in our melanoma mouse model. During late phase, when the tumor is characterized by remodeling of necrotic areas and tumor re-growth, tumor vessels provided by the D849N-mutant host stroma are more densely covered by perivascular cells, but this does not to result in any appreciable tumor growth advantage. During early phases of tumor development however, the mutant host stroma supports the establishment of tumor more efficiently, due to increased vascularization. This is an important observation, as it demonstrates that activating mutations of growth factor receptors in the host stroma can accelerate tumor establishment in the absence of tumor-derived ligand expression, and thus contribute to shortened tumor dormancy.

## Abbreviations

PDGFR, platelet-derived growth factor receptor; IHC, immunohistochemistry

## Competing interests

The author(s) declare that they have no competing interests.

## Authors' contributions

SS participated in the study design, carried out most of the animal studies, performed all of the histological evaluation, including statistical analysis, and drafted the manuscript. CHH was involved in study design and drafting of the manuscript. RLH has conceived of the study, participated in study design and coordination, and drafting of the manuscript. All authors read and approved the final manuscript.

## Pre-publication history

The pre-publication history for this paper can be accessed here:


